# Supplementary material for: Interactions between Human Gut Microbiome Dynamics and Sub-Optimal Health Symptoms during Seafaring Expeditions
Source: Microbiol Spectr. 2022 Jan 12;10(1):e00925-21. doi: 10.1128/spectrum.00925-21 (PMC8754112; doi:10.1128/spectrum.00925-21)
Supplement: SUPPLEMENTAL FILE 1 — Supplemental material. Download SPECTRUM00925-21_Supp_1_seq6.pdf, PDF file, 0.6 MB [file spectrum00925-21_supp_1_seq6.pdf]

Supplementary Materials for

Interactions between human gut microbiome dynamics and sub-optimal health symptoms during seafaring expeditions

The additional tables include:

Table S1. Annotation for microbial species in the co-occurrence network

Table S2. The important score of the 28 microbial species and functions used in the Random Forest model

Table S3. Definitions of the 24 indicators in the questionnaire

Table S4. The questionnaire answers for all individuals before and after the voyage

The supplementary figures include:

Figure S1. Comparison of individual perturbations in the gut microbiome between 'symptomless' and 'symptom' crew members for 24 indicators (symptoms)

Figure S2. Co-occurrence networks of the SS and non-SS at beginning (day 1) and end (day 135) of the voyage

Reference

Table S1. Annotation for microbial species in the co-occurrence network.

Nodes ID	Species name (annotation in mOTUs2)	Nodes ID	Species name (annotation in mOTUs2)
**S0004**	Clostridiales species incertae sedis [meta_mOTU_v25_13280]	**S0716**	Coprobacter fastidiosus [ref_mOTU_v25_03667]
**S0008**	Faecalibacterium prausnitzii [ref_mOTU_v25_06109]	**S0720**	Ruminococcaceae species incertae sedis [meta_mOTU_v25_13151]
**S0019**	Bacteroides cellulosilyticus/fragilis [ref_mOTU_v25_01597]	**S0723**	Eubacterium sp. CAG:180 [ref_mOTU_v25_07675]
**S0030**	Bacteroides plebeius [ref_mOTU_v25_05069]	**S0727**	[Ruminococcus] torques [ref_mOTU_v25_03703]
**S0032**	Clostridiales species incertae sedis [meta_mOTU_v25_13006]	**S0735**	Dorea longicatena [ref_mOTU_v25_03692]
**S0033**	Clostridiales species incertae sedis [meta_mOTU_v25_12707]	**S0736**	Clostridiales species incertae sedis [meta_mOTU_v25_12434]
**S0052**	Ruminococcus bicirculans [ref_mOTU_v25_02792]	**S0749**	Bacteroides coprocola [ref_mOTU_v25_11279]
**S0060**	Coprococcus eutactus [ref_mOTU_v25_02378]	**S0752**	[Eubacterium] hallii [ref_mOTU_v25_03632]
**S0082**	Proteobacteria sp. [ref_mOTU_v25_00095]	**S0754**	Dorea longicatena [ref_mOTU_v25_03693]
**S0085**	Clostridiales species incertae sedis [meta_mOTU_v25_13168]	**S0756**	Bacteroides coprophilus [ref_mOTU_v25_04094]
**S0093**	Clostridiales species incertae sedis [meta_mOTU_v25_12253]	**S0761**	Faecalibacterium prausnitzii [ref_mOTU_v25_06108]
**S0094**	Firmicutes sp. [ref_mOTU_v25_03629]	**S0763**	Clostridium sp. CAG:127 [meta_mOTU_v25_12306]
**S0100**	Clostridiales species incertae sedis [meta_mOTU_v25_12356]	**S0766**	Bacteroides finegoldii [ref_mOTU_v25_03474]
**S0103**	Sutterella sp. CAG:351 [meta_mOTU_v25_13411]	**S0769**	Prevotella sp. CAG:279 [meta_mOTU_v25_12279]
**S0114**	Blautia obeum/wexlerae [ref_mOTU_v25_02154]	**S0770**	Roseburia inulinivorans [ref_mOTU_v25_00860]
**S0115**	Clostridium sp. AT4 [meta_mOTU_v25_12609]	**S0772**	Firmicutes sp. [ref_mOTU_v25_02374]
**S0116**	Paraprevotella xylaniphila [ref_mOTU_v25_03699]	**S0777**	Roseburia sp. CAG:303 [meta_mOTU_v25_12911]
**S0117**	Pasteurellaceae species incertae sedis [meta_mOTU_v25_13253]	**S0781**	Eubacterium sp. CAG:156 [ref_mOTU_v25_07673]
**S0124**	Clostridiales species incertae sedis [meta_mOTU_v25_12252]	**S0785**	Clostridiales species incertae sedis [meta_mOTU_v25_12265]
**S0127**	Bifidobacterium pseudocatenulatum [ref_mOTU_v25_02700]	**S0792**	Prevotella stercorea [ref_mOTU_v25_00717]
**S0129**	[Ruminococcus] gnavus [ref_mOTU_v25_01594]	**S0794**	Clostridium sp. CAG:492 [meta_mOTU_v25_12462]
**S0134**	Ruminococcaceae species incertae sedis [meta_mOTU_v25_12664]	**S0804**	Oscillibacter sp. 57_20 [meta_mOTU_v25_12282]
**S0144**	Paraprevotella clara [ref_mOTU_v25_03698]	**S0812**	Roseburia sp. 40_7 [meta_mOTU_v25_12352]
**S0146**	Eubacterium species incertae sedis [meta_mOTU_v25_12474]	**S0814**	[Clostridium] clostridioforme/bolteae [ref_mOTU_v25_03442]
**S0148**	Bacteroides massiliensis [ref_mOTU_v25_02135]	**S0837**	Clostridiales species incertae sedis [meta_mOTU_v25_12301]
**S0151**	Clostridiales species incertae sedis [meta_mOTU_v25_12873]	**S0840**	Firmicutes bacterium CAG:227 [ref_mOTU_v25_07734]
**S0155**	Ruminococcaceae species incertae sedis [meta_mOTU_v25_12259]	**S0842**	Roseburia sp. [ref_mOTU_v25_03650]
**S0156**	Clostridiales species incertae sedis [meta_mOTU_v25_12452]	**S0850**	Clostridiales species incertae sedis [meta_mOTU_v25_12361]
**S0164**	uncultured Butyricicoccus sp. [ref_mOTU_v25_07314]	**S0853**	Ruminococcaceae species incertae sedis [meta_mOTU_v25_12239]
**S0167**	Clostridium sp. CAG:217 [meta_mOTU_v25_12270]	**S0867**	Clostridiales species incertae sedis [meta_mOTU_v25_12288]
**S0191**	[Ruminococcus] torques [ref_mOTU_v25_03659]	**S0868**	Clostridiales species incertae sedis [meta_mOTU_v25_12229]
**S0197**	Butyricicoccus species incertae sedis [meta_mOTU_v25_12336]	**S0874**	Veillonella parvula [ref_mOTU_v25_01938]
**S0198**	Streptococcus sp. [ref_mOTU_v25_00283]	**S0881**	Clostridiales species incertae sedis [meta_mOTU_v25_13200]
**S0204**	Oscillibacter sp. [ref_mOTU_v25_03341]	**S0883**	Odoribacter splanchnicus [ref_mOTU_v25_03697]
**S0207**	Prevotella species incertae sedis [meta_mOTU_v25_12332]	**S0890**	Holdemanella biformis [meta_mOTU_v25_12329]
**S0211**	Clostridiales species incertae sedis [meta_mOTU_v25_12522]	**S0899**	Bacteria sp. [ref_mOTU_v25_00077]
**S0214**	Bifidobacterium adolescentis [ref_mOTU_v25_02703]	**S0907**	Bacteroides stercoris [ref_mOTU_v25_01579]
**S0229**	Clostridiales species incertae sedis [meta_mOTU_v25_12251]	**S0916**	Clostridiales species incertae sedis [meta_mOTU_v25_12476]
**S0236**	Enterobacteriaceae sp. [ref_mOTU_v25_00096]	**S0924**	Alistipes putredinis [ref_mOTU_v25_03683]
**S0240**	Eubacterium sp. CAG:38 [ref_mOTU_v25_07679]	**S0932**	Clostridiales species incertae sedis [meta_mOTU_v25_13187]
**S0247**	Ruminococcus sp. CAG:177 [meta_mOTU_v25_12400]	**S0939**	Haemophilus influenzae/parainfluenzae [ref_mOTU_v25_01068]
**S0253**	Holdemanella biformis [ref_mOTU_v25_11382]	**S0952**	Roseburia species incertae sedis [meta_mOTU_v25_12366]
**S0255**	Bacteroides caccae [ref_mOTU_v25_03473]	**S0959**	Bifidobacterium ruminantium [ref_mOTU_v25_02702]
**S0257**	Eubacterium sp. CAG:274 [meta_mOTU_v25_12688]	**S0967**	Blautia massiliensis [ref_mOTU_v25_03342]
**S0262**	Eubacterium ramulus [ref_mOTU_v25_03570]	**S0970**	Clostridiales species incertae sedis [meta_mOTU_v25_14166]
**S0264**	Alistipes finegoldii [ref_mOTU_v25_03682]	**S0971**	Clostridium species incertae sedis [meta_mOTU_v25_12350]

**S0269**	Flavonifractor plautii [ref_mOTU_v25_02971]	**S0972**	uncultured Flavonifractor sp. [ref_mOTU_v25_07315]
**S0271**	Acinetobacter sp. [ref_mOTU_v25_03673]	**S0973**	Flavonifractor plautii [ref_mOTU_v25_05238]
**S0272**	Firmicutes sp. [ref_mOTU_v25_03641]	**S0974**	Alistipes shahii [ref_mOTU_v25_01014]
**S0274**	Bacteroidales species incertae sedis [meta_mOTU_v25_13286]	**S0981**	Bacteroides faecis/thetaiotaomicron [ref_mOTU_v25_01657]
**S0277**	Butyricicoccus species incertae sedis [meta_mOTU_v25_12617]	**S0994**	Bacteroides salyersiae [ref_mOTU_v25_02140]
**S0279**	Bifidobacterium longum [ref_mOTU_v25_01099]	**S1006**	Bacteroides fragilis [ref_mOTU_v25_01598]
**S0287**	Ruminococcus lactaris [ref_mOTU_v25_01595]	**S1016**	Clostridiales species incertae sedis [meta_mOTU_v25_13390]
**S0303**	Clostridium sp. CAG:230 [ref_mOTU_v25_07616]	**S1018**	Clostridiales species incertae sedis [meta_mOTU_v25_12463]
**S0305**	Clostridiales species incertae sedis [meta_mOTU_v25_12631]	**S1019**	Clostridium sp. CAG:7 [ref_mOTU_v25_07647]
**S0306**	Blautia obeum [ref_mOTU_v25_05140]	**S1021**	Collinsella aerofaciens [ref_mOTU_v25_03626]
**S0308**	Clostridium sp. [ref_mOTU_v25_03622]	**S1023**	Sutterella wadsworthensis [ref_mOTU_v25_04772]
**S0310**	Burkholderiales species incertae sedis [meta_mOTU_v25_12785]	**S1034**	Eubacterium ventriosum [ref_mOTU_v25_11151]
**S0321**	Lachnospiraceae species incertae sedis [meta_mOTU_v25_12240]	**S1043**	Roseburia intestinalis [ref_mOTU_v25_03702]
**S0327**	Clostridium species incertae sedis [meta_mOTU_v25_12399]	**S1047**	Clostridium sp. CAG:138 [meta_mOTU_v25_12233]
**S0344**	Eubacterium sp. CAG:202 [meta_mOTU_v25_12308]	**S1052**	Prevotella sp. CAG:520 [ref_mOTU_v25_00718]
**S0357**	Roseburia hominis [ref_mOTU_v25_00861]	**S1053**	Alistipes ihumii [ref_mOTU_v25_07379]
**S0363**	Oscillibacter sp. [ref_mOTU_v25_04664]	**S1059**	Clostridiales species incertae sedis [meta_mOTU_v25_12411]
**S0367**	Clostridium sp. CAG:58 [ref_mOTU_v25_07640]	**S1065**	Clostridiales sp. [ref_mOTU_v25_03634]
**S0389**	Faecalibacterium species incertae sedis [meta_mOTU_v25_12303]	**S1066**	Clostridiales species incertae sedis [meta_mOTU_v25_12653]
**S0400**	Faecalibacterium species incertae sedis [meta_mOTU_v25_12325]	**S1067**	Bacteroides caecimuris [ref_mOTU_v25_03476]
**S0401**	Streptococcus parasanguinis [ref_mOTU_v25_00312]	**S1069**	Clostridiales species incertae sedis [meta_mOTU_v25_12554]
**S0403**	Clostridiales species incertae sedis [meta_mOTU_v25_12742]	**S1070**	Ruminococcaceae species incertae sedis [meta_mOTU_v25_12264]
**S0406**	Romboutsia timonensis [ref_mOTU_v25_09389]	**S1075**	Clostridiales species incertae sedis [meta_mOTU_v25_12635]
**S0421**	Ruminococcus callidus [ref_mOTU_v25_11153]	**S1078**	Bacteroidales species incertae sedis [meta_mOTU_v25_13098]
**S0433**	Coprococcus catus [ref_mOTU_v25_11851]	**S1082**	Clostridium sp. CAG:302 [meta_mOTU_v25_12499]
**S0436**	Clostridiales species incertae sedis [meta_mOTU_v25_13377]	**S1091**	Firmicutes species incertae sedis [meta_mOTU_v25_12231]
**S0437**	Eubacterium species incertae sedis [meta_mOTU_v25_12314]	**S1092**	Ruminococcus sp. CAG:254 [meta_mOTU_v25_12276]
**S0440**	Clostridiales species incertae sedis [meta_mOTU_v25_12473]	**S1102**	Clostridiales species incertae sedis [meta_mOTU_v25_14118]
**S0448**	Streptococcus sp. [ref_mOTU_v25_01350]	**S1114**	Fusobacterium mortiferum [ref_mOTU_v25_11278]
**S0450**	Clostridium sp. CAG:299 [ref_mOTU_v25_07619]	**S1122**	Firmicutes bacterium CAG:102 [ref_mOTU_v25_07726]
**S0452**	Faecalibacterium prausnitzii [ref_mOTU_v25_06110]	**S1123**	Parabacteroides merdae [ref_mOTU_v25_05401]
**S0488**	Parabacteroides distasonis [ref_mOTU_v25_03640]	**S1142**	Clostridiales species incertae sedis [meta_mOTU_v25_12309]
**S0489**	Alistipes obesi [ref_mOTU_v25_06695]	**S1145**	Firmicutes bacterium CAG:114 [ref_mOTU_v25_07728]
**S0493**	Clostridiales species incertae sedis [meta_mOTU_v25_12563]	**S1149**	Ruminococcus bromii [ref_mOTU_v25_00853]
**S0512**	[Clostridium] leptum [ref_mOTU_v25_03688]	**S1159**	Clostridiales sp. [ref_mOTU_v25_03658]
**S0519**	Anaerostipes hadrus [ref_mOTU_v25_00857]	**S1160**	Ruminococcus species incertae sedis [meta_mOTU_v25_12766]
**S0520**	Blautia obeum [ref_mOTU_v25_05139]	**S1161**	Bacteroides dorei/vulgatus [ref_mOTU_v25_02367]
**S0526**	Sutterella wadsworthensis [ref_mOTU_v25_03066]	**S1167**	Faecalibacterium species incertae sedis [meta_mOTU_v25_12403]
**S0548**	[Eubacterium] eligens [ref_mOTU_v25_02376]	**S1168**	Bacteroides intestinalis [ref_mOTU_v25_02809]
**S0551**	Allisonella histaminiformans [ref_mOTU_v25_10689]	**S1186**	Clostridium species incertae sedis [meta_mOTU_v25_12324]
**S0562**	Clostridiales species incertae sedis [meta_mOTU_v25_13227]	**S1187**	Clostridiales species incertae sedis [meta_mOTU_v25_12786]
**S0572**	Clostridium sp. [ref_mOTU_v25_03680]	**S1190**	Bacteroidales species incertae sedis [meta_mOTU_v25_12262]
**S0578**	Coprococcus sp. [ref_mOTU_v25_01683]	**S1191**	Clostridiales species incertae sedis [meta_mOTU_v25_12674]
**S0585**	Alistipes finegoldii/onderdonkii [ref_mOTU_v25_03069]	**S1193**	unclassified
**S0588**	Barnesiella intestinihominis [ref_mOTU_v25_03595]	**S1194**	Clostridiales sp. [ref_mOTU_v25_03605]
**S0590**	Dialister sp. CAG:357 [meta_mOTU_v25_12661]	**S1208**	Oscillibacter sp. 57_20 [meta_mOTU_v25_12610]
**S0596**	Eubacterium sp. [ref_mOTU_v25_03633]	**S1209**	Faecalibacterium sp. [ref_mOTU_v25_03636]
**S0602**	Blautia hansenii [ref_mOTU_v25_03670]	**S1215**	Coprococcus comes [ref_mOTU_v25_03690]
**S0606**	Clostridium species incertae sedis [meta_mOTU_v25_12762]	**S1225**	[Eubacterium] eligens [ref_mOTU_v25_02375]

**S0610**	Clostridium species incertae sedis [meta_mOTU_v25_12640]	**S1227**	Dorea formicigenerans [ref_mOTU_v25_03668]
**S0615**	Bacteroides eggerthii [ref_mOTU_v25_01577]	**S1232**	Clostridiales species incertae sedis [meta_mOTU_v25_12556]
**S0622**	Ruminococcaceae species incertae sedis [meta_mOTU_v25_12286]	**S1243**	Ruthenibacterium lactatiformans [ref_mOTU_v25_04716]
**S0653**	Enterobacterales sp. [ref_mOTU_v25_00085]	**S1248**	Clostridiales species incertae sedis [meta_mOTU_v25_12437]
**S0657**	[Eubacterium] rectale [ref_mOTU_v25_03657]	**S1257**	Clostridiales species incertae sedis [meta_mOTU_v25_12284]
**S0663**	Clostridiales species incertae sedis [meta_mOTU_v25_12776]	**S1259**	bacterium LF-3 [ref_mOTU_v25_03628]
**S0677**	Clostridiales species incertae sedis [meta_mOTU_v25_13012]	**S1264**	Anaerostipes hadrus [ref_mOTU_v25_00856]
**S0679**	Bacteroides sp. [ref_mOTU_v25_03475]	**S1265**	Phascolarctobacterium succinatutens [ref_mOTU_v25_03700]
**S0681**	Faecalibacterium prausnitzii [ref_mOTU_v25_06112]	**S1267**	Eubacterium species incertae sedis [meta_mOTU_v25_12260]
**S0684**	Bacteroides rodentium/uniformis [ref_mOTU_v25_00855]	**S1276**	Ruminococcus sp. [ref_mOTU_v25_03681]
**S0698**	Clostridiales species incertae sedis [meta_mOTU_v25_13863]	**S1277**	Oscillibacter species incertae sedis [meta_mOTU_v25_12353]
**S0699**	Prevotella copri [ref_mOTU_v25_03701]	**S1287**	Bacteroides sp. [ref_mOTU_v25_02810]
**S0703**	Bilophila wadsworthia [ref_mOTU_v25_04300]	**S1289**	[Ruminococcus] torques [ref_mOTU_v25_03660]
**S0709**	Bifidobacterium bifidum [ref_mOTU_v25_03116]	**S1293**	Firmicutes species incertae sedis [meta_mOTU_v25_12744]
**S0710**	Parasutterella excrementihominis [ref_mOTU_v25_03674]	**S1294**	Megamonas funiformis/rupellensis [ref_mOTU_v25_02318]
**S0711**	Clostridium species incertae sedis [meta_mOTU_v25_12234]		

Table S2. The important score of the 28 microbial species and functions used in the Random Forest model.

ID	Name	Importance	Mean for those with ocean-going syndrome	mean for the 'symptomless' group
**K17076**	Environmental Information Processing; Membrane transport; ABC transporters; lysY; putative lysine transport system ATP-binding protein	0.0102	7.71E-05	1.43E-04
**K17074**	Environmental Information Processing; Membrane transport; ABC transporters; lysX2; putative lysine transport system permease protein	0.0100	8.06E-05	1.48E-04
**K17073**	Environmental Information Processing; Membrane transport; ABC transporters; lysX1; putative lysine transport system substrate-binding protein	0.0117	7.16E-05	1.35E-04
**K11928**	Brite Hierarchies; Protein families: signaling and cellular processes; Transporters; putP; sodium/proline symporter	0.0104	3.50E-04	4.39E-04
**K10118**	Environmental Information Processing; Membrane transport; ABC transporters; msmF; raffinose/stachyose/melibiose transport system permease protein	0.0095	8.21E-04	1.11E-03
**K09815**	Environmental Information Processing; Membrane transport; ABC transporters; znuA; zinc transport system substrate-binding protein	0.0104	2.02E-04	2.65E-04
**K07727**	Brite Hierarchies; Protein families: genetic information processing; Transcription factors; putative transcriptional regulator	0.0044	4.69E-04	5.05E-04
**K07080**	Not Included in Pathway or Brite; Poorly characterized; Function unknown; uncharacterized protein	0.0192	1.40E-04	2.09E-04
**K06379**	Not Included in Pathway or Brite; Unclassified: metabolism; Enzymes with EC numbers; spoIIAB; stage II sporulation protein AB (anti-sigma F factor)	0.0157	4.52E-04	5.42E-04
**K06188**	Brite Hierarchies; Protein families: signaling and cellular processes; Transporters; aqpZ; aquaporin Z	0.0027	7.33E-05	5.51E-05
**K04023**	Not Included in Pathway or Brite; Unclassified: metabolism; Amino acid metabolism; eutH; ethanolamine transporter	0.0057	4.73E-05	1.87E-05
**K03496**	Brite Hierarchies; Protein families: genetic information processing; Chromosome and associated proteins; parA, soj; chromosome partitioning protein	0.0060	2.76E-03	3.11E-03
**K03466**	Brite Hierarchies; Protein families: genetic information processing; Chromosome and associated proteins; ftsK, spoIIIE; DNA segregation ATPase FtsK/SpoIIIE, S-DNA-T family	0.0149	2.39E-04	3.63E-04
**K03458**	Not Included in Pathway or Brite; Unclassified: signaling and cellular processes; Transport; TC.NCS2; nucleobase:cation symporter-2, NCS2 family	0.0061	1.30E-04	1.85E-04
**K02662**	Brite Hierarchies; Protein families: signaling and cellular processes; Secretion system; pilM; type IV pilus assembly protein PilM	0.0061	8.92E-05	1.29E-04
**K02408**	Cellular Processes; Cell motility; Flagellar assembly; fliE; flagellar hook-basal body complex protein FliE	0.0046	1.96E-04	2.42E-04
**K01787**	Metabolism; Carbohydrate metabolism; Amino sugar and nucleotide sugar metabolism; RENBP; N-acylglucosamine 2-epimerase	0.0023	1.43E-04	1.93E-04
**K01775**	Metabolism; Metabolism of other amino acids; D-Alanine metabolism; alr; alanine racemase	0.0062	4.99E-04	5.50E-04
**K01278**	Organismal Systems; Digestive system; Protein digestion and absorption; DPP4, CD26; dipeptidyl-peptidase 4	0.0006	1.61E-05	2.35E-05
**K01182**	Metabolism; Carbohydrate metabolism; Galactose metabolism; IMA, malL; oligo-1,6-glucosidase	0.0060	3.74E-04	3.84E-04
**K00865**	Metabolism; Carbohydrate metabolism; Glyoxylate and dicarboxylate metabolism; glxK, garK; glycerate 2-kinase	0.0037	5.23E-04	4.79E-04
**K00806**	Metabolism; Glycan biosynthesis and metabolism; Peptidoglycan biosynthesis; uppS; undecaprenyl diphosphate synthase	0.0152	6.21E-04	7.59E-04
**K00171**	Metabolism; Carbohydrate metabolism; Glycolysis / Gluconeogenesis; porD; pyruvate ferredoxin oxidoreductase delta subunit	0.0047	3.15E-05	2.72E-05
Eubacterium sp. CAG 192	*Eubacterium sp. CAG 192*	0.0016	0.00E+00	5.33E-06
uncultured Blautia sp.	*uncultured Blautia sp.*	0.0025	3.96E-05	1.74E-05
Eubacterium ventriosum	*Eubacterium ventriosum*	0.0059	1.35E-03	7.88E-04
Actinomyces viscosus oris	*Actinomyces viscosus oris*	0.0050	2.64E-06	1.53E-05

Table S3. Definitions of the 24 indicators in the questionnaire.

Indicator	Definition
Body mass index (BMI)	The BMI is defined as the body mass divided by the square of the body height (1).
Backache	Back pain, also known as backache, is pain felt in the back (2).
Headache	It is usually confined to the upper part of the head, including the upper edge of the eyebrow arch, the helix and the pain above the line connecting the external occipital protuberance (3).
Stomachache	Stomach pain refers to the pain in the epigastric part of the upper abdomen, above the connection line between the lower edges of ribs on both sides and near the heart socket under the xiphoid process (4).
Pectoralgia	Pectoralgia is a subjective feeling, feeling chest pain, severe pain, dull pain, stuffy pain or oppressive feeling (5).
Muscular soreness	Pain or tenderness in one or more muscles which can involve any area of the body.
Dyspnea	Dyspnea is a combination of subjective feeling and objective signs. Subjectively, breathing is laborious, objectively, the number of breaths increases, the movement is fast and the range increases (6).
Palpitation	Palpitation includes palpitation and palpitation, which is a kind of uncomfortable feeling or palpitation of conscious heart beating (7).
Insomnia	Insomnia is medically defined as being unable to fall asleep or remain asleep, resulting in insufficient sleep (8).
Shallow sleep	Difficulty in falling asleep, decreased sleep quality and sleep time.
Over-eating	Overeating means taking a huge amount of food in a very short time (9).
Nausea	Nausea is a kind of gastric discomfort that can cause vomiting impulse (10).
Inactivity	People's mental and physical abilities are not as good as before, and will feel tired when they engage in daily activities (11).
Poor appetite	Lack of appetite or lack the desire to eat (12).
Feelings of loneliness	Loneliness is an unpleasant emotional response to perceived isolation (13).
Depression	Depression (mood), a state of low mood and aversion to activity (14).
Self-accusation	Self-accusation is a cognitive process in which an individual attributes the occurrence of a stressful event to oneself (15).
Mistrustfulness	Mistrustfulness is an individual's distrust of other people's words, promises and statements (16).
Stool type	Type 1: Separate hard lumps (like nuts), or sausage-shaped (but lumpy); Type 2: Like a sausage but with cracks on its surface (can be black);Type 3: Like a sausage or snake, smooth and soft (average stool) Type 4: Soft blobs with clear cut edges; Type 5: A mushy stool or watery (diarrhoea) (17).
Bloody stools	The color of the stool is bright red, dark red or tar like (black stool) (18).
Incomplete defecation	This term is a feeling of incomplete defecation. When defecating, even if the intestinal contents have been emptied, they still want to be discharged.
Bowel difficulties	The defecation was laborious, painful and prolonged.
Bowel movement	Want to defecate but can't row out, or squat down for a long time to row out (19).
Defecation frequency (weekly)	Frequency of defecation per week. The normal frequency of bowel movements in adults is between three per day and three per week (20).

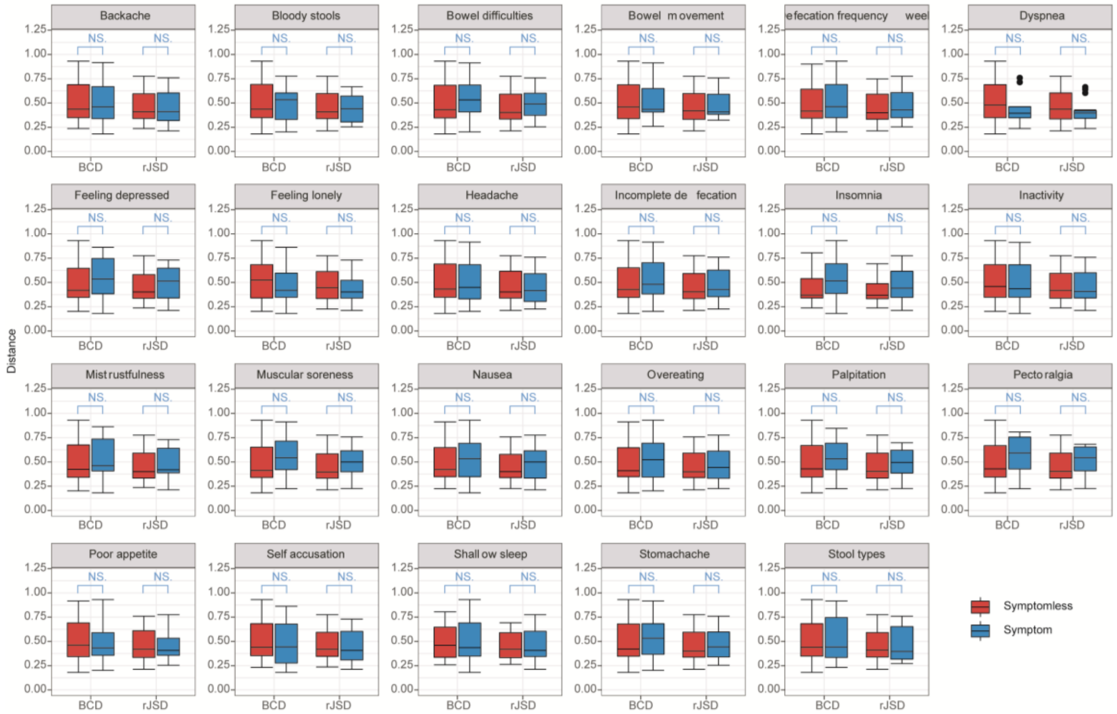

Figure S1. Comparison of individual perturbations in the gut microbiome between 'symptomless' and 'symptom' crew members for 24 indicators (symptoms). Individual perturbations (n=77) in the gut microbiome measured by BCD and rJSD were first grouped according to whether they were 'symptomless' or 'symptom' for each individual health indicator (symptom), and then compared with each other using Wilcoxon rank-sum Test.

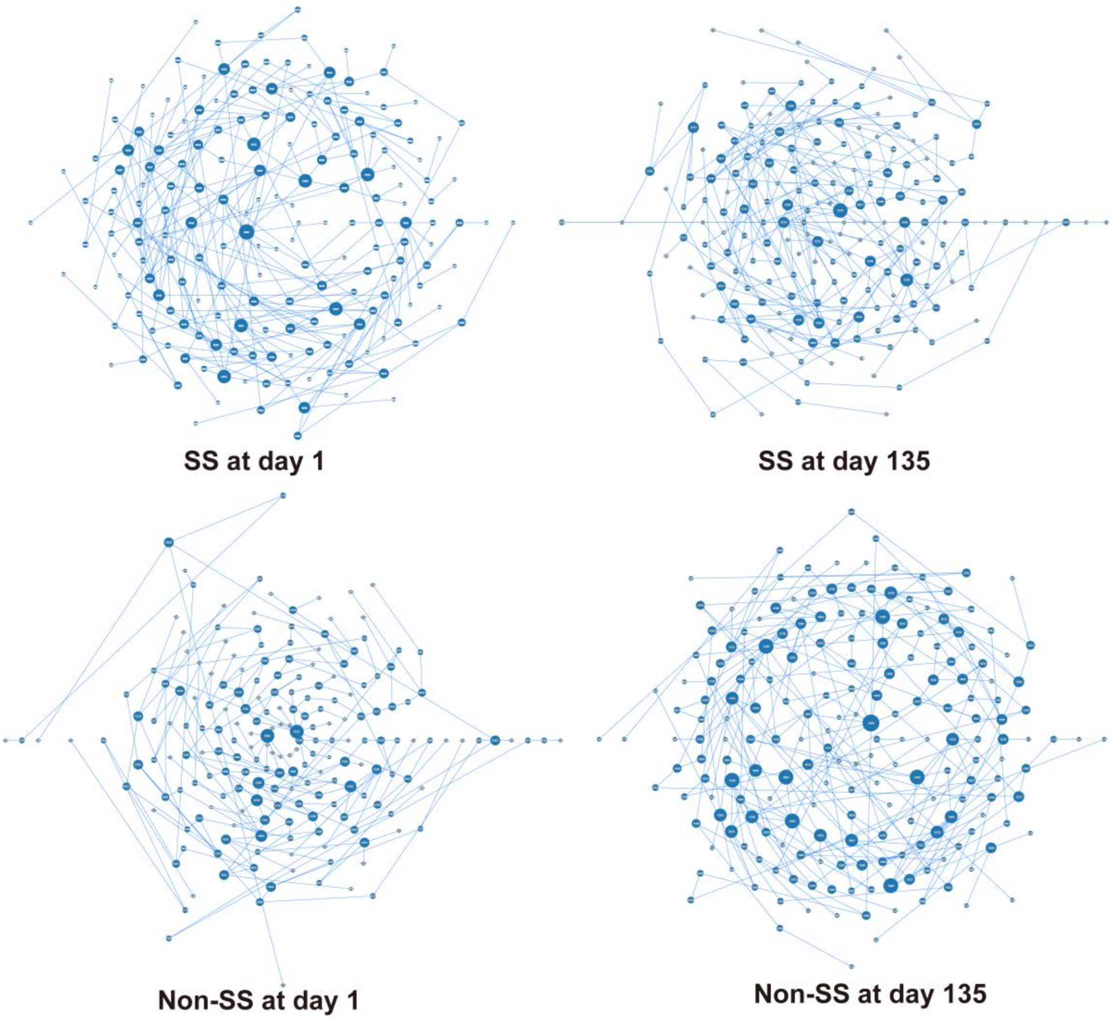

Figure S2. Co-occurrence networks of SS and non-SS at beginning (day 1) and end (day 135) of the voyage. Four co-occurrence networks of SS and non-SS at the beginning (day 1) and end (day 135) of the voyage are separately illustrated, which are based on SparCC correlation inference algorithms. The nodes in the network refer to the identified species with the size of the nodes indicating their relative abundance; The edges between nodes represent correlations between the nodes they connect.

REFERENCES

1. Anonymous. 2016. Body Mass Index (BMI) Charts. Pediatr Dent 38:418-419.
2. Casazza BA. 2012. Diagnosis and treatment of acute low back pain. Am Fam Physician 85:343-50.
3. Rizzoli P, Mullally WJ. 2018. Headache. Am J Med 131:17-24.
4. Viniol A, Keunecke C, Biroga T, Stadje R, Dornieden K, Bösner S, Donner-Banzhoff N, Haasenritter J, Becker A. 2014. Studies of the symptom abdominal pain--a systematic review and meta-analysis. Fam Pract 31:517-29.
5. Horn B. 2015. [Chest pain]. Ther Umsch 72:62-5.
6. Anonymous. 1999. Dyspnea. Mechanisms, assessment, and management: a consensus statement. American Thoracic Society. Am J Respir Crit Care Med 159:321-40.
7. Giada F, Raviele A. 2018. Clinical Approach to Patients with Palpitations. Card Electrophysiol Clin 10:387-396.
8. Buysse DJ. 2013. Insomnia. Jama 309:706-16.
9. Engin A. 2017. Eat and Death: Chronic Over-Eating. Adv Exp Med Biol 960:53-80.
10. Metz A, Hebbard G. 2007. Nausea and vomiting in adults--a diagnostic approach. Aust Fam Physician 36:688-92.
11. Sharara E, Akik C, Ghattas H, Makhlouf Obermeyer C. 2018. Physical inactivity, gender and culture in Arab countries: a systematic assessment of the literature. BMC Public Health 18:639.
12. van der Meij BS, Wijnhoven HAH, Lee JS, Houston DK, Hue T, Harris TB, Kritchevsky SB, Newman AB, Visser M. 2017. Poor Appetite and Dietary Intake in Community-Dwelling Older Adults. J Am Geriatr Soc 65:2190-2197.
13. Tabue Teguo M, Simo-Tabue N, Stoykova R, Meillon C, Cogne M, Amiéva H, Dartigues JF. 2016. Feelings of Loneliness and Living Alone as Predictors of Mortality in the Elderly: The PAQUID Study. Psychosom Med 78:904-909.
14. Cui R. 2015. Editorial: A Systematic Review of Depression. Curr Neuropharmacol 13:480.
15. Janoff-Bulman R. 1979. Characterological versus behavioral self-blame: inquiries into depression and rape. J Pers Soc Psychol 37:1798-809.
16. Greben SE. 1984. The re-establishment of trust through psychotherapy. Can J Psychiatry 29:350-4.
17. Mínguez Pérez M, Benages Martínez A. 2009. The Bristol scale - a useful system to assess stool form? Rev Esp Enferm Dig 101:305-11.
18. Anonymous. 1990. *In* Walker HK, Hall WD, Hurst JW (ed), Clinical Methods: The History, Physical, and Laboratory Examinations. Butterworths Copyright © 1990, Butterworth Publishers, a division of Reed Publishing., Boston.
19. Holl RM. 2014. Bowel movement: the sixth vital sign. Holist Nurs Pract 28:195-7.
20. Panigrahi MK, Kar SK, Singh SP, Ghoshal UC. 2013. Defecation frequency and stool form in a coastal eastern Indian population. J Neurogastroenterol Motil 19:374-80.
